# Punishing to Send a Message ^†^

**DOI:** 10.1093/ojls/gqaf034

**Published:** 2025-10-13

**Authors:** Angelo Ryu, Trenton Sewell

**Keywords:** punishment, consequentialism, retributivism, expressivism, blame

## Abstract

In *Punishment for the Greater Good*, Adam Kolber defends consequentialism as a better justification for punishment than retributivism. Here, we reject the dichotomy and seek to motivate expressivism as a genuine alternative. According to expressivism, what justifies punishment is its expression of a fitting message. We show how expressivism can be developed to avoid Kolber's objections to retributivism, while having a number of advantages over his preferred consequentialism.

## 1. Introduction

Some argue criminal punishment is never justifiable in principle, even after idealising the particulars of its implementation. Others allow that it is potentially justifiable. Among them, two families of views are prominent. There are many problems with this crude division, but they go by the labels of ‘consequentialism’ and ‘retributivism’. Roughly, consequentialists defend punishment as a way of achieving better overall outcomes while retributivists say it gives criminals what they deserve.

Neither label easily fits the justification of punishment as a fitting expressive response to criminal conduct. The state punishes, on this view, to send a valuable message. Call this expressivism about punishment.

A recent monograph provides the occasion to revisit expressivism. This is so despite—or, indeed, because of—the marginal role it plays in the book. In *Punishment for the Greater Good*, Adam Kolber argues for the superiority of a specific kind of consequentialism (‘pure consequentialism’) over a specific kind of retributivism (‘standard retributivism’). Missing from Kolber's picture is expressivism. We argue this is a mistake.

The plan is as follows. The second section lays out the book's general structure. The third section introduces expressivism and contrasts it with pure consequentialism and standard retributivism. The fourth section considers Kolber's central objection to retributivism. As it turns out, the objection also poses a challenge to pure consequentialism. Expressivism avoids it, however. The next three sections examine Kolber's other arguments for pure consequentialism over standard retributivism. The same pattern arises. By his own lights, expressivism is preferable to pure consequentialism. Before concluding, the eighth section addresses an important objection to expressivism.

## 2. The Dichotomy

The book is framed around two positions, which serve to answer a distinctive question.

First, the positions. Kolber describes them as pure consequentialism (PC) and standard retributivism (SR). PC is Kolber's own preferred position. In short, it holds that we should maximise good consequences, with what makes a consequence ‘good’ mostly left open. SR, by contrast, has more content. It affirms axiological retributivism, which Kolber defines as a commitment to there being ‘intrinsic value’ in making wrongdoers suffer (or be subject to some other form of harsh treatment) for their conduct.^1^ He then identifies as paradigmatic of SR two additional core beliefs and three supplementary features.^2^ The two beliefs are:

- Deserved punishment is proportional to the seriousness of wrongdoing.
- The state should never intentionally, or knowingly, inflict more punishment than what is deserved.

And the three features are:

- A deontological moral theory, according to which the rightness of actions may sometimes correspond to worse overall consequences.
- The recognition of criminal defendants having certain fundamental rights, such as a right not to be convicted absent a fair trial where guilt is shown beyond a reasonable doubt.
- A partial concession to consequentialism … such that in at least some situations the goodness of consequences is relevant to right action when both retributivism and deontological principle are silent.

Second, the question. What question does PC and SR seek to answer? Much work in the philosophy of punishment is framed in the register of ideal theory. It asks what justifies the punishment practice we might hope to have in a just society where crime still regrettably occurs. Kolber, by contrast, is interested in whether PC or SR is better at justifying punishment in the here and now.^3^ That is, how do they fare given the limits of our present situation? He concludes PC is preferable.

Chapter 1 of the book lays out the two positions and the question Kolber hopes to answer. Chapter 2 provides some background on consequentialist ethics and endorses positions which will be familiar to utilitarians. Chapter 3 argues that consequentialism can explain various intuitions that are widely believed to undermine it. These intuitions include, for instance, that it is wrong to torture someone, even if doing so would save several lives. Such intuitions, Kolber says, are best understood as ‘heuristics’ or ‘decision shortcuts’ under conditions of epistemic uncertainty.^4^ Chapter 4 builds on this, arguing that various cases often taken to embarrass consequentialism—such as whether the state ought to punish the innocent for the greater good—may actually be *more* embarrassing for retributivists. The chapter's strategy is to present a dilemma to SR: either endorse absolute deontology, which Kolber takes to be absurd, or retreat to threshold deontology, which he rejects as unacceptably arbitrary.^5^

The first four chapters flesh out, and defend, PC. While the fourth chapter criticises SR, it is largely defensive in tone. It focuses on matters of normative ethics largely abstracted from issues particular to punishment. In the next two chapters, by contrast, Kolber seeks to turn retributivism against itself.

Chapter 5 seeks to show that requiring proportionate punishment commits SR to unintuitive results. Chapter 6 targets the Blackstone ratio, namely that ‘it is better that ten guilty persons escape than that one innocent suffer’.^6^ Kolber argues, due to uncertainty arising from our epistemic limitations, that there is ‘too much uncertainty for standard retributivists to actually punish’ consistent with the Blackstone ratio.^7^

Finally, chapter 7 discusses abolitionism. Kolber thinks we should, ideally, do away with punishment. But given the value it serves in the here and now, he concludes we cannot get rid of it just yet.

## 3. Expressivism

Kolber considers two positions: pure consequentialism and standard retributivism. They represent leading efforts to justify punishment. But there is a third family of views, distinct from the two he focuses on: expressivism.

What separates punishment from, say, a burdensome tax? Joel Feinberg gives an intriguing answer: ‘Punishment is a conventional device for the expression of attitudes of resentment and indignation, and of judgements of disapproval and reproportion … Punishment, in short, has a *symbolic significance* largely missing from other kinds of penalties.’^8^

Some, like Feinberg, only mean this as a conceptual claim regarding what punishment is. For others, however, it is a crucial step in the justification of punishment.^9^ Wrongdoers warrant punishment, on this view, much like how the blameworthy warrant blame. The expression inherent in punishment is a fitting response to criminal conduct. We therefore have reason to impose it.

Not only is punishment, understood as expressed condemnation, apt given the wrongdoer's conduct, it may be necessary to respect the values the wrongdoer transgresses. The state disavows the wrong, and in this respect refuses to acquiesce in it.^10^ By ‘going on the record’, the state assures its people that the law is ‘vindicated’.^11^ ‘To remain silent in the face of crime would be to betray the values which the law expresses, and to which we are committed.’^12^ It acknowledges the victim was injured through the fault of another, as opposed to being an accident.^13^ Absent punishment, the state would fail to recognise the crime's ‘wrongful character’.^14^ Within the expressive paradigm, punishment is understood to censure, and is therefore partly justified on the non-consequentialist ground of it being an intrinsically apt response.

On this picture, the offender is specially situated respecting the state's need to send the relevant message. The crime gives rise to the circumstances under which the state must disavow the act.^15^ The state must now, as a matter of principle, reaffirm its commitment to the values, interests and rights that underpin the criminal offence.

Crucially, expressivism is neither PC nor SR. The contrast with PC is straightforward: the expressivist reason to punish an offender for a crime concerns not the good consequences *going forward*, but the aptness of the message punishment expresses *given what occurred*.

The way expressivism differs from SR is more nuanced. The notion of ‘desert’, characteristic of retributivist thought, features in many expressivist views. This raises an important question: is desert necessary for expressivism as such or does it play a more contingent role? But we will set this aside for a moment, as there is a clear sense in which expressivism is distinct from SR *as Kolber characterises it*. This is because expressivism is not committed to axiological retributivism, namely the position that the suffering or hard treatment endured by wrongdoers, if deserved, is intrinsically valuable. On the expressivist picture, it is valuable to express the relevant message, such as censure. It does not follow that all aspects of punishment—like the infliction of suffering or the deprivation of liberty—are themselves intrinsically valuable. Although hard treatment is part of punishment, it is the expression that is intrinsically valuable, not how the expression is achieved.^16^

Further, the content of what is expressed need not cash out in retributive terms. The message sent could be something other than ‘D, having φed, deserves to suffer’. There is disagreement among expressivists about which precise message the state should express. But the message could take the non-retributive form of affirming the wrongness and culpability of the wrongdoer's conduct, recognising the victim's moral status, insisting on the state's commitment to certain values, and so on.^17^

Expressivism is therefore distinct from PC or SR. Still, you may wonder whether expressivism is a genuinely third theory of punishment. Can it be reduced to either consequentialism or retributivism, once they are construed more loosely than PC and SR? This is a difficult question, for, once we move away from Kolber's tightly defined positions, it becomes less clear what defines their respective boundaries.

Some expressivist theories look like retributivism with a special currency. On such views, wrongdoers deserve censure rather than hard treatment.^18^ Other views, however, do not invoke desert. Rather, they focus on wrongdoers rendering themselves liable to being punished to send a message.^19^ Now you may suppose any view concerned with liability is necessarily a species of retributivism. After all, that the liability of wrongdoers arise from their desert is one way to understand the position sometimes known as ‘negative’ retributivism.^20^ At the same time, seeing retributivism in this way might broaden the camp beyond the point of theoretical fruitfulness.^21^

Moreover, some expressivists invoke neither desert nor liability.^22^ Rather, they argue it is permissible to punish the guilty to the extent our reasons to send the message defeat our reasons not to punish. And the guilty may be specially situated, irrespective of any liability, since the expressive value of punishment only arises when the guilty, rather than the innocent, are punished. This would be the case, for instance, if the expressive value only arises from the *apt* expression of the relevant message.

A diverse range of views are therefore recognisably expressivist. Some are largely consequentialist, while others mostly travel along classical retributivist lines. Still, expressivism can be understood as a distinctive approach, in ways setting it apart from most consequentialist and retributivist views. The focus is on punishment's expressive character. The message it conveys plays a crucial role in its justification.

Here, we argue that the distinctive aspects of expressivism, taken mostly on their own, raise it above PC and SR. This is consistent with it ultimately being a species of retributivism rather than an alternative to it. Our interest is not, primarily, in a categorisation exercise. There are many ways to define consequentialism and retributivism. For precisely this reason, Kolber focuses his attention on what he defines as PC and SR. For Kolber, they specify, with greater rigour, the distinctive commitments of two ways to justify punishment. But his dichotomy leaves out expressivism. This is regrettable, we argue, since expressivism warrants attention as a serious contender.

Before we proceed, a quick word on what we do *not* seek to achieve. We will not be able to provide a full defence of a particular expressivist theory of punishment. What we aim to accomplish, assuming we succeed in our two objectives, is more modest. First, to motivate the further development of expressivism as an alternative to PC and SR. And second, to show how expressivism might be developed, consistent with its core commitments, in ways responsive to Kolber's important arguments.

## 4. Here and Now

Kolber argues that PC is better situated than SR to justify punishment in the here and now. This frames the inquiry in terms of how a given official—working within a particular practice, faced with all sorts of practical constraints such as scarcity of time and attention—might justify the incarceration of a given defendant.

Put this way, the issue is not how to justify the existence of a system of punishment in the abstract. Here, Kolber breaks from one traditional conceptualisation of the point of punishment theory.^23^ According to it, the primary issue is whether we should have a certain social practice. What favours the punishment of a given person might be affected by what justifies the practice. But the practice's justification has, in some sense, priority over the justification of the punishments composing the practice.

Kolber rejects this, and we agree. To justify a practice of punishment in the here and now is to justify the token instances of punishment comprising the practice. Our focus should be on the ‘moral justification of particular actions’.^24^ That justification must then speak to the discrete choices of officials operating within the system. For instance: ‘Should I, a juror, dismiss this indictment that could otherwise lead to jail time? Should I … vote to convict this defendant’?^25^ What is centrally in need of justification is the punishment of a specific person to a specific degree on a specific occasion.

This, however, poses a serious challenge to Kolber's own view, for certain phenomena on which PC might seek to appeal, such as deterrence, are largely insensitive to tokens of punishment.

Plausibly, deterrence involves both positive and negative general prevention. Positive prevention reduces the incidence of crime by reinforcing social norms inconsistent with crime.^26^ Negative prevention reduces the incidence of crime by making it prudentially unwise. This is consistent with supposing that deterrence, understood as a diffuse phenomenon, primarily consists of the social conditions under which ‘people do not develop inclinations to offend’.^27^ On this view, deterrence works by ‘inculcating a culture in which people do not form intentions to do wrong’.^28^ Deterrence, so understood, cannot help PC justify punishment in the here and now. Consider:

**Situation A.** There is a fully functioning justice system in which roughly 100,000 people are punished each year. This year, one possible candidate for punishment is Ava. She faces sentencing for hiring a hit man on Blake.

Understood as a one-off deviation, letting Ava get off without punishment is unlikely to lower general deterrence. Which individuals will form wrongful intentions when they otherwise would not? The benefits of deterrence are overdetermined at the social level, since any token of punishment is insignificant in its marginal effect on general deterrence. Now consider:

**Situation B.** Everything is the same as Situation A, except Ava never hires a hit man, such that she is not a possible candidate for punishment.

Assume the state punishes every eligible candidate. This means one fewer token of punishment in Situation B. If Ava's punishment as such contributes to deterrence, we would expect the crime rate to go up, however minimally. This is because, in Situation B, there are fewer opportunities to bolster the prudential reinforcement of anti-crime inclinations.^29^ But this is implausible.

To be sure, a conspicuous failure to punish Ava might undermine deterrence. For it could encourage the inference that, in a sufficiently wide range of cases, the state will not punish. Punishing Ava is significant, on this view, not to secure a marginal benefit to deterrence, but to avoid a loss.

This is contingent on a number of factors, however. First, Ava's case needs to have widespread public salience. Many cases go under the radar.^30^ Second, the state can likely block the inference even while omitting punishment for Ava. For instance, it could give reasons for the failure which are particular to Ava. Or, alternatively, the omission could arise from a rare exercise of the pardon power. There are, in short, ways to make clear the omitted punishment is a ticket good for one ride only.

We therefore conclude the marginal benefit of an added token of punishment is minimal or non-existent. PC must therefore appeal to consequences other than deterrence to justify punishment. An obvious alternative is incapacitation. Like deterrence, this pertains to crime prevention. However, it solely concerns the need to stop wrongdoers from committing crimes during the period of imprisonment. This is only valuable if the wrongdoer would otherwise reoffend, and is only justified if the value of stopping the crimes they would otherwise commit outweigh the costs of locking them up. It seems to follow that any crime less serious than kidnapping would be unjustified on PC. Some may like this conclusion, but it fails by Kolber's own lights as a justification of punishment in the here and now.

Expressivists straightforwardly avoid this implication since they justify punishment by appeal to backward-looking reasons. The need to send a message refers to the wrong *having been committed*, which makes fitting the state's condemnation of it. At the same time, expressivists need not deny that the practice of punishment has an overall deterrent effect on crime. This consequentialist value may be crucial to the explanation of our social commitment to punishing. It could therefore be an important consideration for a *system designer* who must decide whether the state should be in the business of punishing, even if it is largely irrelevant to a *judge* who must justify the punishment of a particular defendant in the here and now.

## 5. Innocents

A serious worry with PC is its seeming inability to explain the wrong of punishing the innocent. Chapters 3 and 4 offer a response.

Chapter 3 argues PC is consistent with the intuitive wrongness of innocent punishment. Kolber says the intuition derives from various heuristics (‘decision shortcuts’) that are useful under conditions of empirical uncertainty.^31^ If so, it simply serves as a rule of thumb; innocents should usually be left unpunished. A worry with this strategy is that it leaves the objection untouched, for it concedes innocent punishment is acceptable so long as the consequences add up.

Chapter 4 addresses this point. Kolber says SR has it worse on this front. This is because SR must either commit to an implausibly absolutist prohibition on punishing the innocent or endorse a mysterious ‘threshold’ after which the deontological restriction falls away.

Many standard retributivists find an absolute prohibition on innocent punishment, no matter how high the cost, to be implausible. This leads them to the second option. Doing so, Kolber says, moves the goalposts.

After criticizing consequentialists for allowing the punishment of the innocent, threshold deontologists end up allowing the same thing. For both theories, when bad consequences are sufficiently dire, both consequentialists and (non-absolutist) retributivists allow the knowing punishment of the innocent. Apparently, we’re just haggling over the price at which we discard somewhat parallel principles (called constraints or prohibitions for retributivists and called shortcuts for consequentialists).^32^

Kolber does not think the ‘haggling’ is neutral between PC and SR. This is because, as he puts it, threshold deontologists ‘leave mysterious the location of their thresholds and have no obvious method of deciding when deontological prohibitions are superseded by consequentialist considerations’.^33^ The charge, in other words, is that the threshold is unacceptably vague and *ad hoc*.

To sum up, Kolber has two objections to threshold retributivists. First, they cannot hold against PC the bare possibility of punishing the innocent, since they also concede it is sometimes permissible. Second, the location of the threshold is arbitrary.

Respecting the first objection, there is at least one respect in which threshold retributivists fare better than pure consequentialists, for PC is committed to an implausible symmetry between guilty and innocent punishment. Say that each inflicts precisely the same degree of suffering. If so, the justificatory burden of innocent and guilty punishment is identical. What is meant to make the difference is that punishing the innocent is often harmful in ways punishing the guilty is not. Conversely, punishing the guilty tends to bring about certain goods which do not ordinarily arise when punishing the innocent. Still, these are contingent differences. The burden of justification is fundamentally symmetrical.

Even if threshold retributivists cannot rule out innocent punishment, they can still insist such punishment is different in kind, morally speaking, from guilty punishment. Punishing the innocent infringes a right they possess, however justifiably. Punishing the guilty does not. The difference, in other words, is between the existence of a defeated right and there not being a right at all. This captures an important aspect of the relevant phenomenology. There is something special to regret with innocent punishment, since in such cases there is always a person whose right is infringed.

Kolber is aware of this response. He rejects it, saying consequentialists can also have ‘residual regrets and concerns’.^34^ It is perfectly consistent to maximise the good while regretting that the maximal achievable good is not even higher. Similarly, consequentialists can minimise harm while regretting the harm that inevitably arises.

This response does not work. The pure consequentialist might, while punishing the innocent, regret not being able to do better. But the same regret could arise while punishing the guilty. The pure consequentialist could, for instance, regret that the guilty must suffer pain, even if that pain is necessary for the greater good. The problem, in short, is that PC treats the reasons for regret as symmetrical across guilty and innocent punishment. The two are asymmetrical, even if there are extraordinary occasions in which innocent punishment is permissible.

Still, Kolber may well be correct in his rejection of threshold deontology as unacceptably arbitrary. A full defence of it would require a generalised account of how to fix the location of its decisive threshold, which we do not provide here.^35^ Rather, we seek to show how expressivism can, absent reliance on threshold deontology, vindicate the intuition that guilty and innocent punishment are asymmetrical.^36^

According to expressivism, punishing wrongdoers for φing sends a certain message. That message, whatever else it says, expresses that the wrongdoer is guilty of φing. When innocents are punished, such that they have not φed (or are not culpable for φing), something is seriously amiss. The punishment is not fitting; it is not apt. Innocents are falsely represented.

This is a familiar thought in work on blame and culpability. There, a common thought is that, for it to be fitting or apt to blame *S* for φing, the appraisal involved in blame must accurately represent *S*.^37^ Since expressivism extends this approach to punishment, it naturally vindicates an asymmetry between guilty and innocent punishment. This is because, no matter how good our reasons to punish the innocent, they can never make it *fitting* to punish them. Even if unfitting punishment is exceptionally permissible, the cause for regret is ineliminable and unique.

## 6. Proportionality

SR is committed to punishment only being permissible if proportionate to the crime. Many see this as a good-making feature of retributivism. Chapter 5 turns this around. There, Kolber seeks to show how it is, actually, ‘more a liability than an asset’ for SR.^38^ For retributivists, there is something—call it *X*—that must exist in proportion to the wrongdoer's culpability for φing. Kolber's basic strategy is to show that, however we define X, unintuitive conclusions follow.

First, Kolber considers equating *X* with punishment. On this view, the offender's punishment must be proportionate to culpability. This is a natural view. But once we specify what we mean by punishment, Kolber thinks it becomes unacceptable.

A common thought is that punishment ‘must be intentionally imposed’.^39^ What, then, of the following case? Suppose two defendants are equally culpable for the same act. Both are convicted. The two come before difference judges for sentencing. Each judge imposes a prison term of equal length. One judge, however, has intimate knowledge of prisons, having previously used to work as a prison guard. She intends ‘not only that inmates be deprived of their liberties of motion but that they also have limited opportunities to see family, have sex, express themselves’, and so on.^40^ The other judge, by contrast, was previously a bankruptcy lawyer. She only has a vague understanding of prisons. She intends only to limit the defendant's freedom of movement. If punishment depends on intention, the two judges impose drastically different punishment, which is unintuitive. And if punishment is *X*, then at least one defendant is either over- or under-punished.

Another problem with *X* being punishment is credit for pretrial detention. Such detention is not meant as punishment. Although it does lead to hard treatment, this is meant only as a regrettable byproduct of a necessary precaution, not an intentional infliction. If *X* is punishment, it would then be mysterious why time served in pretrial detention could ever make a later punishment disproportionate.^41^

Next, Kolber considers equating *X* not with punishment, but with hard treatment. This variant of SR requires what defendants actually endure to be proportionate to the crime. But Kolber argues this also faces insurmountable problems. He focuses on three. Each follows the same pattern. Take two equally blameworthy defendants, sentenced to prison terms of equal length. Kolber identifies three ways in which, if *X* were hard treatment, seemingly irrelevant facts are made to bear on proportionality.

*Different facilities.* One defendant is sent to a cushy prison while the other goes someplace more austere. For their treatment to be proportionate, it seems that the prisoner subject to the worse conditions must spend less time in prison. This becomes implausible once we specify why certain prisoners are sent to worse prisons. Even among those who are equally culpable for their past conduct, there is variance in how much danger they pose going forward. In light of this, officials ‘generally assign more dangerous inmates to higher-security prisons with more austere conditions’.^42^ The costly conclusion is that the more dangerous prisoner must be released earlier.

*Different subjective experiences.* Two defendants could ‘vary substantially in their experiences of confinement, often predictably so’.^43^ For example, they may be differently sensitive to the same conditions. One could be claustrophobic while the other is not. The wealthy, being used to luxurious conditions, may well be more sensitive in this respect. But it seems unacceptable to punish the privileged less severely on this basis.^44^

*Different baselines.* Normally, we assess harm compared to some baseline. Sometimes, the baseline is historical: harm arises when we are worse off than before. Other times, the baseline is counterfactual: harm arises when we are worse off than we would have been had the injury not occurred. ‘The key point is that harm is a worsening from one reference point to another.’^45^ Whether that point is defined in historical or counterfactual terms, certain defendants will have a much higher baseline than others. This means the same prison sentence could lead to dramatically different degrees of harm. For instance, the wealthy have a higher baseline, so that they suffer more than from being subject to identical conditions.

There is some awkwardness in Kolber's discussion of these factors. We agree their relevance to punishment is unintuitive. They thus pose a challenge to SR. It is objectionable in principle for the rich, given their subjective experience or harm baseline, to be treated more leniently than the poor. But that is precisely what PC requires, for it assesses punishment against the harm inflicted on the prisoner. It follows that, if wealthy prisoners suffer greater harm, then more is needed to justify their punishment compared to the poor. In short, Kolber's objections do not uniquely target SR's proportionate punishment requirement. They apply to any view that takes the extent of harm on the prisoner to directly bear on the justifiability of punishment.

To be sure, PC could tell a story of how, due to public order (or something like it), the state must *pretend* that the rich are harmed equally as the poor. The greater good, in other words, could require the state to act as if the wealthy and poor are relevantly equivalent. We are not sure there is such a story, however, and in any event Kolber does not provide one.

None of this, of course, rescues SR, for it still seems more vulnerable to these problems than PC. Kolber drives home this point by rigorously assessing, and rejecting, several other ways to modify SR to potentially make the proportionality requirement more acceptable.^46^ Then he briefly discusses how PC explains some of our intuitions regarding the significance of proportionality.^47^

The problems Kolber points out with proportionality do not apply to expressivism, however. This is because, although proportionality matters to expressivism, it plays a very different role than in SR. For SR, the proportionality relation is essentially a brute metaphysical or moral matter. The suffering wrongdoers ought to receive happens to stand in some definable mathematical relation to their crime's seriousness. But why think X must be ‘proportionate’ to the crime rather than, say, Y?

This is not how expressivists typically conceive of proportionality. For them, what must be proportionate to the offence is the *censure* expressed by punishment. This makes relevant the extent of hard treatment.^48^ If the state inflicts too much punishment, it sends too strong a message about the defendant's conduct. If the state punishes too little, it sends too weak a message. The requirement of proportionate hard treatment corresponds to the need for wrongdoers to endure a certain amount of hard treatment for the right message to be expressed.^49^

This means that, even if Judge A intends to impose double the disadvantage as Judge B, both typically send the same message when giving a sentence of equal length. This is because, in our community, a sentence's length serves a symbolic role in conveying the relevant message. The private mental states of judges are irrelevant to the content of the message, even if they bear on the analytic question of whether something counts as punishment.

Along similar lines, expressivism can straightforwardly address at least two of Kolber's objections to proportionate treatment, namely differences in subjective experience and baseline. While such differences alter the amount of hard treatment an offender experiences, this need not correspond to a difference in the expressed message.^50^ So expressivists can explain why we do not ordinarily reduce sentences in ways we might otherwise expect under PC or SR. The wealthy receiving shorter prison sentences, for instance, sends the wrong message about the severity of crimes committed by the rich.

What about different facilities? The issue, recall, is that two equally culpable defendants could be subject to vastly different prison conditions. It follows that, even with a sentence of equivalent length, the extent of suffering might substantially diverge. As it turns out, however, expressivism can give an appropriately nuanced answer to this challenge.

Sometimes, a difference in facilities has no symbolic significance bearing on the message conveyed. Other times, however, the difference matters to the expressed censure. Compare, for instance, being sent to Guantanamo Bay as opposed to a standard prison. Even if for a shorter period, it is plausible this expresses greater condemnation.

While Kolber largely neglects expressivist theories, chapter 5 briefly discusses a neighbouring view. Referring to Alon Harel and Richard Frase,^51^ Kolber considers whether a focus on punitive expression could make the subjective experience of punishment irrelevant, since one could ‘simply understand punishment in purely objective terms’.^52^ Kolber does not outline how this argument might proceed, but it is likely to resemble the expressivist response we have sketched so far.

Kolber rejects the argument. He argues it elides the difference between caused harm and perceived harm. As he puts it:

If all nonincarcerated Samoans forget that the country skipped a day on the calendar, it wouldn't ease the actual severity of the sentences of inmates confined on the day that was skipped. More dramatically, if the public believes that people of a certain race do not feel pain, those mistaken beliefs carry no weight when assessing whether some harm can be justifiably inflicted. Similarly, merely believing that a day in prison inflicts the same harm on all inmates doesn't make it so.^53^

There are two ideas here. First, the public symbolism of an act does not, alone, alter the amount of an offender's suffering. Second, how the public regards some punishment should not alter the quantum of permissible punishment.

We will not question the first idea. But the second fails to take expressivism seriously. If proportionality is understood along SR lines as a deep normative fact about the world, then the suggestion that it depends on public attitudes becomes deeply implausible. This is simply not how it works for expressivists, however. On expressivism, *of course* public attitudes matter. Take a non-legal example. Say two people are arguing at dinner. One of them throws wine in the other's face and storms out. This sends a certain message, potentially warranted—but only given the context, which imbues the act with the relevant meaning.

At the same time, expressivists can affirm that the cases Kolber has in mind are highly problematic. For instance, imposing a sentence higher than the legally permitted amount, as in the Samoan case, violates the rule of law; and defendants have a right against racist treatment, irrespective of proportionality. Put another way, these cases are objectionable for reasons external to a theory of punishment. There are many more ways for punishment to be impermissible than for it to be justified.

## 7. Risk

In the here and now, officials must punish under conditions of uncertainty. Kolber exploits this fact to raise an objection to SR, which he lays out in chapter 6.

Many jurisdictions require the prosecution to clear a high evidential bar before punishment is permissible. For instance, a conviction in the United States requires proof beyond a reasonable doubt (BARD). It looks like SR can neatly explain this feature of our practice. The high evidential bar corresponds to an asymmetry between guilty and innocent punishment. This corresponds to Blackstone's suggestion that ‘it is better that ten guilty persons escape than that one innocent suffer’.^54^

Although this feature of SR appears to be a strength, chapter 6 seeks to undermine it. To see how, note that BARD only concerns whether the elements of a crime are satisfied. This, however, is not enough for those in the business of punishment: they must be sufficiently confident that all the *moral requirements* for permissible punishment are met. Put another way, the asymmetry between guilty and innocent punishment, and therefore BARD, outstrips the question of whether all the elements of a crime are satisfied. The heightened standard of justification should extend to proof that the punishment is, in all respects, morally justified.

If this is correct, officials must be confident that a series of additional conditions are met if they are to justifiably punish under SR. These include the existence of free will; the general possibility of moral responsibility; punishment being an appropriate response to wrongs; and the absence of other principled objections that might potentially take punishment off the table. These conditions, which bear on the permissibility of punishment as a general matter, are controversial. They are also only some of what must be true for punishment to be justified by retributivism's own lights. Moving to conditions specific to a given punishment, retributivists must believe the defendant has done what is alleged, the alleged conduct is wrongful, the sentence is proportionate, and so on.

The chapter's key challenge is that standard retributivists cannot believe the guilty deserve punishment without placing an unreasonably high credence on the existence of each condition. Say there are nine necessary conditions, each of which we believe are satisfied with 95% confidence. To punish, retributivists must believe the much less likely proposition that all these conditions obtain.^55^ If we assume the likelihood of each condition is independent of one another, we can only be 63% sure that all nine conditions are jointly satisfied. This alone seems inconsistent with the Blackstonian values underlying BARD. Given the value SR places on not punishing the innocent, the degree of risk seems unacceptable. Kolber therefore presents a dilemma. ‘For reasons of consistency, standard retributivists must either rethink their commitment to the BARD standard or rethink their commitment to retributivism.’^56^

There are many potential responses. For instance, retributivists could deny that the probability of each condition is independent. If so, Kolber cannot safely multiply the respective probabilities to arrive at a dramatically lower probability. In response, Kolber insists we should, when assessing the likelihood of each condition, assume the other conditions all exist.^57^ But this complication introduces additional noise in our assessment of likelihood. We must consider our credence not just on a single condition, but conditioned on the existence of other conditions. At the same time, given the complexity of SR, Kolber is right to insist that substantial moral uncertainty will plausibly persist.

Given this, retributivists could restrict the heightened justificatory burden such that it only applies to *some* of the necessary conditions. But then they must explain how the restriction is consistent with the values underlying a heightened standard of justification. A criminal trial, to be sure, only concerns itself with certain issues, such as whether the defendant was unconscious, and not others, such as whether free will exists in general. But this alone does not explain why the legal distinction has any deep moral significance.^58^ What does the legal distinction have to do with the moral asymmetry between guilty and innocent punishment?

It may be that some retributivists conceive of BARD as a tool to reduce moral uncertainty, even if it only applies to certain questions and not others. On this view, the concern is instrumental. The focus is on BARD's ability to reduce overall uncertainty to a tolerable degree. But now BARD seems seriously inadequate. Even if it secures a high credence in the defendant having committed the crime, many more controversial propositions must be true for SR to justify punishment.

By contrast, Kolber says PC faces no such challenges since it denies any fundamental asymmetry between guilty and innocent punishment. This allows consequentialists to say ‘the uncertainty of punishment is counterbalanced by the moral uncertainty of *failing* to punish’.^59^ It follows, however, that PC faces a problem in the other direction: as Kolber accepts, PC lacks a deep explanation for BARD.^60^ At best, it can say that, in the here and now, we should not swiftly jettison it, for this might cause public disruption and undermine the legal system's stability.^61^ Fair enough, but this is a costly implication for those who find BARD, or something like it, intuitive.

Unlike PC, expressivism can explain BARD while avoiding Kolber's moral uncertainty objection. Here, we provide two possible explanations, although there may be others.

First, by holding itself to a high evidential standard, the state expresses moral concern for factual innocence. This, in turn, communicates the significance of treating individuals as a freestanding object of moral concern. To be sure, this is a costly way to express the concern, to the extent it makes successful prosecutions more challenging. But, as we discuss in the next section, the costliness of the message plays a crucial role in bolstering its credibility. By punishing in this way, the state puts its money where its mouth is. It treats the value as sufficiently significant to warrant the cost.

Second, the state, by holding itself to the high evidential standard of BARD, lends credibility to the condemnatory message it conveys about the offender. The difficulty of imposing punishment aids its expressive power, for the very reason that it is hard for the state to punish.

This alone does not determine where to draw the line. Why not have an even higher standard? The answer is that, given the importance of sending a message, there are compelling reasons not to functionally disable the state from making the relevant expression. The criminal standard of proof, then, is the product of a careful dance: it enhances the expressive power of punishment, but at the cost of making it harder for the state to make that expression.

What about the moral uncertainty objection? With an alternative explanation of BARD in hand, we can now lower the justificatory standard for punishment. Recall that the source of SR's vulnerability is its commitment to a stark asymmetry in the significance of avoiding innocent punishment as compared to imposing guilty punishment. Given the force of Kolber's argument, we are prepared to accept that this asymmetry cannot be as substantial as many retributivists have supposed.

This is not *ad hoc*, since expressivists can affirm both that innocents have a *right* not to be used to send a condemnatory message and that the state has a *duty* to censure wrongs. In other words, expressivism is consistent with there being weighty reasons to avoid innocent punishment and impose guilty punishment. This is something expressivism has in common with PC. At the same time, it can reject PC's implausible commitment to innocent and guilty punishment being symmetrical.

The need to express a punitive message, in turn, helps expressivism avoid the moral uncertainty objection. Recall that the challenge is to show why BARD applies to matters of empirical fact but not questions of high philosophical theory, like the problem of free will. The duty to censure wrongs explains this difference. This is because, if BARD were applicable to the latter kind of issue, then it would always be unsatisfied. The state could never punish. This outcome plainly strikes the wrong balance between the need to punish and the need for that punishment to carry sufficient expressive power.

## 8. Hard Treatment

If we are correct, expressivism avoids Kolber's objections against SR while having certain advantages over PC. At the same time, expressivism is not without its problems. Arguably, the most serious is that of hard treatment. When it comes to punishment, this is what is perhaps most in need of justification. How can it be permissible to lock someone up in a dark room? The answer, according to expressivism, lies in the state's need to condemn the wrongdoer's conduct. This is the primary respect in which expressivism departs from SR. According to expressivism, hard treatment is valuable not in itself, but as enabling the expression of a valuable message. What justifies punishment is the value of that message, not the hard treatment.

However, this gives rise to a serious explanatory deficit. Although hard treatment plainly serves the function of expressing condemnation, this is not enough. It can be the case that locking someone up is a way of condemning their wrongful conduct, without it being the only way. Why is *prison* the ‘most appropriate mode of communication’?^62^

We take this to be an important challenge to expressivism. Even if there were a duty to send a message, the question remains why the state must punish to send it. This poses a serious threat to expressivism as a genuine alternative to standard retributivism and pure consequentialism. The worry is that, depending on how it answers the problem of hard treatment, it collapses to a variant of either SR or PC. On one view, even if punishment is characterised by its expressive function, the choice between alternative modes of expression is dictated by the need to prevent crime.^63^ But this makes expressivism a variant of PC. On another view, punishment kills two birds with one stone: it expresses an apt message while giving wrongdoers the hard treatment they deserve. This, however, makes expressivism a variant of SR.

Set these answers aside. Are there any distinctively expressivist justifications of hard treatment? Presently, the leading option is that it enhances the credibility of the expression.

Hard treatment is needed to show that the disapprobation is meant seriously. For example, an academic department does not show disapproval of a serious lapse by a colleague merely through a verbal admonition; to convey the requisite disapproval, some curtailment of privileges is called for.^64^

This is a promising start, but it alone will not do. The problem is the centrality of the role it places on contingent social conventions. On this view, punishment involves hard treatment, since it is the conventional way to express condemnation. That is just how we do things around here.

Without more, this is too contingent to be a satisfactory justification of punishment. The relevant conventions are arbitrary. So, we should simply demand a change in our social conventions. It would be a matter of pressing concern to reform society to enable less harmful means to send the message currently conveyed by hard treatment. This approach, in short, makes the link between expression and hard treatment too contingent for the former to plausibly justify the latter.

A more secure connection is necessary—and we think there is a way to draw it. To see how, it might be worth taking a brief detour to another domain in which messages are expressed.

Male peacocks have a ridiculously showy—and beautiful—tail, or train … But trains are heavy and tough to drag around and maintain. And the biggest, most ornate, trains are the heaviest and most difficult to deal with. They are, in a word, costly. So what's going on? As it turns out, the train's cost contributes directly to its function. That's because the train is what signals the fitness of its bearer. The train conveys valuable information to peahens who need it, information that would otherwise be very hard to fake, namely, that this male is strong and healthy and so would help generate strong and healthy offspring.^65^

The lesson is that messages are more credible when the costs of the signal are ratcheted up. This model of explanation is known as costly signalling theory, as developed by social and natural scientists. The basic thought is that, when it is hard for others to directly assess our message, we can exploit the costliness of a mode of communication to shore up our credibility.

Something similar, we propose, is the best way for expressivists to solve the hard treatment problem. Why is hard treatment needed to send the penal message? Because it makes the infliction of punishment costly—and this cost allows the state to credibly signal its commitment to the relevant values that offenders violate with their criminal conduct.^66^

An obvious objection is that punishment, understood this way, is costly in the wrong way. For costly signalling theory to apply, the punishment must be costly to the *punisher*. But the hardness of treatment imposes a cost to the *punished*. How can this possibly count as a relevant cost?

Our answer relies on a basic fact about punishment: that it is *morally costly*. The state, in imposing hard treatment, takes on massive moral risk. First, it had better be the case that the infringed values are important, for, if not, it is doubtful punishment in response to their infringement could ever be justified. More than that, though, the state must take on an array of additional duties if they are to punish without acting in a patently unacceptable way. For instance, it must afford due process to defendants. It must be careful in how it punishes. And there is a more prosaic cost. The fiscal expense of incarcerating even one additional inmate is eye-watering.

These costs, both moral and fiscal, add up. By confining wrongdoers to a room, the state puts itself in what is effectively a parental role with respect to their life. It assumes onerous duties. And the state purports to do so because the wrongdoers did something warranting such treatment. The values they transgress, the state claims, matter so much that imprisonment is the only fitting response. This message is buttressed by the costs the state is prepared to incur to send the message.

This explains why we cannot easily dispense with hard treatment as part of how punishment sends a message. It is not merely a conventionally specified symbolic device. Rather, it functions as a costly signal securing the message's credibility.

But are there not other ways for the state to send a costly message? Think of a practice of giving massive amounts of compensation to victims and their families, or of launching some new social programme in response to wrongdoing. There are reasons to doubt it will be easy to replace punishment, however. First, these options do not quite capture the *moral* costs the state incurs through punishment. Second, each alternative comes with associated problems. Paying money to victims of crime, for instance, encourages fraud and risks conveying disrespect to the extent it places a monetary price on the wrong.

Suppose, though, that it is possible for other social schemes to send the same message as punishment. If such schemes do not exist in the here and now, officials—like judges—must continue to punish. Still, if there are alternative schemes able to convey the relevant message, you may wonder whether system designers ought to do away with punishment over the long run. This is at least one way to understand abolitionism. Crucially, though, expressivists need not solely rely on expressive value when it comes to justifying our punishment practice at the system-wide level. A general practice of punishment, as distinct from the specific tokens of punishment, could serve valuable social functions, including crime prevention. Given this, system designers may have excellent reasons to select, and maintain, punishment as the means for the state to convey a valuable message. On this view, punishment does double duty: it prevents crime while speaking with a moral voice.^67^

## 9. Conclusion

Kolber poses a series of bracing challenges to standard retributivism. The absolutist prohibition on innocent punishment comes under pressure. The requirement that punishment be proportionate to the crime is problematised. And the commitment to heightened evidentiary standards, such as beyond a reasonable doubt, is put to the test.

But the case they present against standard retributivism is less clear-cut than Kolber supposes. And his preferred position, pure consequentialism, may not always avoid the problems he poses. The picture is complicated.

At the same time, Kolber's arguments provide important clues as to how expressivism should proceed, for they highlight ways to flesh out expressivism that fare better than either side of Kolber's dichotomy. This prospect both motivates expressivism and pushes it to develop in certain directions. Take, for instance, the problem of subjective variance in how wrongdoers experience punishment. Expressivism can make such variance irrelevant, but only if the expressive component of punishment is directed at the victim and the political community more generally, rather than the wrongdoer. And if the relevant expression is understood, not just as censuring the wrongdoer but signalling commitment to certain values, then expressivism can provide an alternative explanation for why we insist on proof beyond a reasonable doubt.

There is much left to address. What is the precise expressive content that punishment conveys? Why is the state's expression of that content valuable? And so on. We presently lack ready answers to these questions, but Kolber has given us ample reason to find out.

## Footnotes

1 For Kolber, the term ‘deserved punishment’ serves as a placeholder for what might instead be framed as ‘deserved suffering’ or ‘deserved deprivation of liberty’ (*Punishment* 6).

2 ibid 6–7.

3 ibid 9–15.

4 ibid 16.

5 ibid 16.

6 William Blackstone, *Commentaries on the Laws of England* *358.

7
*Punishment* 17.

8 Joel Feinberg, ‘The Expressive Function of Punishment’ (1965) 49 Monist 397, 400. Emphasis in the original.

9 Andreas von Hirsch, *Censure and Sanctions* (CUP 1994); Anthony Duff, *Punishment, Communication, and Community* (CUP 2001); Joshua Glasgow, ‘The Expressivist Theory of Punishment Defended’ (2015) 34 Law and Philosophy 601; Bill Wringe, *An Expressivist Theory of Punishment* (Palgrave Macmillan 2016).

10 Feinberg (n 8) 404–8.

11 ibid 406–7.

12 Antony Duff, *Trials and Punishments* (CUP 1986) 236.

13 von Hirsch (n 9) 10.

14 Andreas von Hirsch and Andrew Ashworth, *Proportionate Sentencing* (OUP 2005) 30.

15 Klaus Günther, ‘Criminal Law, Crime and Punishment as Communication’ in Andrew Simester, Antje du Bois-Pedain and Ulfrid Neumann (eds), *Liberal Criminal Theory* (Hart Publishing 2014) 131–3.

16 Duff, a leading expressivist, suggests his view is non-retributive for similar reasons: Antony Duff, ‘Penal Communications’ (1996) 20 Crime and Justice 1, 46.

17 Differences among expressivists about the content of the punitive expression also correspond to differences in the relevant non-consequentialist reason to send the message. For Hampton, punishment expresses reaffirmation of the victim's moral status. The state has reason to send this message in response to a crime as it would otherwise leave unaddressed the wrongdoer's expression, namely the denial of the victim's moral status: Jean Hampton, ‘The Retributive Ideal’ in Jean Hampton and Jeffrie Murphy (eds), *Forgiveness and Mercy* (CUP 1988); Jean Hampton, ‘Correcting Harms Versus Righting Wrongs’ (1992) 39 UCLA L Rev 1659. For Wringe, punishment expresses a condemnatory message to society respecting the wrongdoer, which the state has reason to make to the extent it ought to denounce the wrongdoer's conduct: Wringe, *An Expressivist Theory of Punishment* (n 9); Bill Wringe, ‘Rethinking Expressive Theories of Punishment’ (2017) 174 Philosophical Studies 681. For Duff, punishment engages wrongdoers in a moral conversation in which they are held to account, with the aim of inducing full recognition of their wrongful conduct. On this view, the state has reasons *owed to wrongdoers* to express the relevant message: Antony Duff, ‘Communicative Theory’ in Jesper Ryberg (ed), *The Oxford Handbook of the Philosophy of Punishment* (OUP 2024) 90. See also Andrew Lichter, ‘Wrongdoer-Related Reasons for Blame’ (2025) 135 Ethics 489. For Bennett, the focus is similarly on the interests of wrongdoers. But he arrives at a different message, namely the penance wrongdoers would show if they were appropriately sorry for their conduct: Christopher Bennett, *The Apology Ritual* (CUP 2008).

18 Some call this ‘communicative retributivism’: see Alec Walen, ‘Retributivism Justice’ in Edward N Zalta and Uri Nodelman (eds), *The Stanford Encyclopedia of Philosophy* (Winter 2023 edn).

19 Glasgow (n 9) 620–2, 627.

20 JL Mackie, ‘Morality and the Retributive Emotions’ (1982) 1 Criminal Justice Ethics 3; JL Mackie, ‘Retributivism: A Test Case for Ethical Objectivity’ in Joel Feinberg and Hyman Gross (eds), *Philosophy of Law* (4th edn, Wadsworth Publishing 1991) 677; John Braithwaite and Philip Pettit, *Not Just Deserts* (OUP 1990) 34–5; Duff, *Punishment, Communication, and Community* (n 9) 11–12.

21 This would, for example, classify Tadros's view as retributivist: Victor Tadros, *The Ends of Harm* (OUP 2011). For Tadros, the state should punish wrongdoers to prevent crime. This appears consequentialist. But he argues the state is only permitted to punish wrongdoers for the greater good *if* they have rendered themselves liable to be used for the purposes of crime prevention in virtue of their wrongdoing. So, while the notion of ‘liability’ is central to Tadros's view, it plays a structurally different role than in standard retributive theories. To be clear, this difference extends to rights forfeiture theories, which are generally understood as a species of negative retributivism: see Christopher Wellman, *Rights Forfeiture and Punishment* (OUP 2017). For Tadros, no rights are forfeited. Rather, wrongdoers incur duties of compensation, which triggers an exception to a deontological prohibition against instrumentalisation.

22 This is a possible reading of several expressivist views: see Wringe, ‘Rethinking Expressive Theories’ (n 17); Bennett (n 17).

23 John Rawls, ‘Two Concepts of Rules’ (1955) 64 Philosophical Review 3; HLA Hart, ‘Prolegomenon to the Principles of Punishment’ (1959) 60 Proceedings of the Aristotelian Society 1.

24
*Punishment* 11.

25 ibid 21.

26 See Andrew von Hirsch and Tatjana Hörnle, ‘Positive Generalprãvention und Tadel’ (1995) 142 Goltdammer's Archiv für Strafecht 261.

27 Tadros (n 21) 281.

28 ibid 282.

29 cf Kimberly Brownlee, ‘What Are the Duties in the Duty View?’ (2012) 5 Jerusalem Rev Leg Stud 62.

30 cf Tadros (n 21) 273.

31
*Punishment* 16.

32
*Punishment* 119.

33 ibid 119.

34 ibid 118.

35 An alternative strategy could be to concede arbitrariness but argue it is less problematic than PC: see Jasper Ryberg, ‘Consequentialism, Threshold Retributivism, and Moral Intuitions’ Criminal Law and Philosophy (forthcoming).

36 Apart from what we say, expressivists could simply include a threshold deontological restriction to vindicate the asymmetry. For instance, the guilty might be *liable* to be used to send a message while the innocent might have a threshold deontological right against it. Here, our aim is solely to illustrate how expressivism can vindicate the asymmetry even after it jettisons resort to the resources of threshold deontology.

37 Those who agree include David Shoemaker, *The Architecture of Blame and Praise* (OUP 2024) ch 6; Matt King, *Simply Responsible* (OUP 2023) ch 4; Gideon Rosen, ‘The Alethic Conception of Moral Responsibility’ in Randolph Clarke, Michael McKenna and Angela Smith (eds), *The Nature of Moral Responsibility* (OUP 2015) ch 3. Others, however, are more sceptical: see Randolph Clarke and Piers Rawlings, ‘True Blame’ (2023) 101 Australasian Journal of Philosophy 736; Robert Pál-Wallin, ‘In Opposition to Alethic Views of Moral Responsibility’ Southern Journal of Philosophy (forthcoming).

38
*Punishment* 129.

39 ibid 129.

40 ibid 132.

41 One worry is that pretrial detention poses a challenge to SR and expressivism alike. This is because the message expressed by pretrial detention is arguably different from the one expressed by post-conviction detention. But unlike SR, it is possible to develop expressivism in directions that avoid this concern. True, if one sees the relevant expression as an act of communication with the wrongdoer in which the state blames the wrongdoer for their conduct and demands remorseful recognition, then the message cannot extend to pretrial detention. If the relevant expression is construed differently, however—such as signalling the state's commitment to certain values which the relevant criminal prohibition protects—then it may be possible to explain credit for pretrial detention on a unified basis with post-trial imprisonment.

42
*Punishment* 140.

43 ibid 140.

44 Douglas Husak, ‘Already Punished Enough’ (1990) 18 Philosophical Topics 79, 82.

45
*Punishment* 147.

46 ibid 152–65.

47 ibid 165–7.

48 von Hirsch (n 9) 17–19.

49 This arrives at ordinal proportionality, namely a scale of what crimes should be punished more than others and what the ratio between various punishments should be. Expressivism arrives at this species of proportionality to the extent there is a relation between the degree of hard treatment and the magnitude of expressed censure. This leads to a common worry: can expressivism also arrive at cardinal proportionality? That is, can it fix, in non-contrastive terms, the amount of punishment proper for a particular defendant? See von Hirsch and Ashworth (n 14) 141–2. While this might pose a serious challenge if we were designing a scheme of punishment on a blank slate, it is not salient once a practice is already up and running, for there are already certain modes of treatment carrying expressive significance. This means, in the here and now, we can arrive at cardinal answers from ordinal assessments given our existing practice.

50 To be more precise, this is one instance in which Kolber's objections favour a particular way for expressivism to develop. Some expressivists, like Duff, view punishment as communication directed at the *wrongdoer*. Others, like Hampton and Wringe, say it is directed at the *community*. On the wrongdoer-directed view, subjective experience and baseline will likely bear on the wrongdoer's uptake of the censure. It therefore seems vulnerable to Kolber's objections. By contrast, on the community-directed view, the problematic factors—to the extent they are insufficiently public or fail to carry the relevant social meaning—will not bear on punishment's expressive content. This point therefore favours community-directed variants of expressivism. Although we do not mean to rule out wrongdoer-directed views, we take this issue to bear on how expressivism should proceed.

51 Alon Harel, ‘Economic Analysis of Law: A Survey’ in Alon Harel and Keith Hylton (eds), *Research Handbook on the Economics of Criminal Law* (Edward Elgar Publishing 2012) ch 1; Richard Frase, *Just Sentencing* (OUP 2012) 110, fn 5.

52
*Punishment* 144.

53 ibid 144.

54 Blackstone (n 6).

55
*Punishment* 174.

56 ibid 177.

57 ibid 191.

58 ibid 201.

59 ibid 205. Emphasis in the original.

60 ibid 207.

61 ibid 207.

62 Duff, *Trials and Punishments* (n 12) 240.

63 ibid 241; von Hirsch (n 9) ch 2.

64 von Hirsch (n 9) 12. Here, von Hirsch relies on the work of Kleinig and Primoratz: John Kleinig, ‘Punishment and Moral Seriousness’ (1991) 25 Israel Law Review 401; Igor Primoratz, ‘Punishment as Language’ (1989) 64 Philosophy 187.

65 Shoemaker (n 37) 28.

66 We refer to values as a useful shorthand, but we mean to include interests, rights, and so on.

67 cf James Edward and Andrew Simester, ‘Prevention with a Moral Voice’ in Simester, du Bois-Pedain and Neumann (n 15) ch 3.

